# Grouping by Proximity in Haptic Contour Detection

**DOI:** 10.1371/journal.pone.0065412

**Published:** 2013-06-07

**Authors:** Krista E. Overvliet, Ralf Th. Krampe, Johan Wagemans

**Affiliations:** Laboratory of Experimental Psychology, University of Leuven (KU Leuven), Leuven, Belgium; Bielefeld University, Germany

## Abstract

We investigated the applicability of the Gestalt principle of perceptual grouping by proximity in the haptic modality. To do so, we investigated the influence of element proximity on haptic contour detection. In the course of four sessions ten participants performed a haptic contour detection task in which they freely explored a haptic random dot display that contained a contour in 50% of the trials. A contour was defined by a higher density of elements (raised dots), relative to the background surface. Proximity of the contour elements as well as the average proximity of background elements was systematically varied. We hypothesized that if proximity of contour elements influences haptic contour detection, detection will be more likely when contour elements are in closer proximity. This should be irrespective of the ratio with the proximity of the background elements. Results showed indeed that the closer the contour elements were, the higher the detection rates. Moreover, this was the case independent of the contour/background ratio. We conclude that the Gestalt law of proximity applies to haptic contour detection.

## Introduction

The Gestalt theory of perceptual organization aims to explain how the brain processes and organizes the incoming stream of perceptual information. Since the early works on the Gestalt theory [1], [2], [3], a large body of research has been conducted on the applicability of the Gestalt principles in visual perception [4], [5]. However, the exact nature of underlying mechanisms remained unclear. A fundamental question that is still unanswered is at which stage of perceptual processing Gestalt principles are operating. The few studies which addressed this issue did not produce clear-cut conclusions: Gestalt principles may be operating at multiple levels with either feed-forward or feedback connections or both [6]. Investigating the applicability of the Gestalt principles in another sensory modality could shed more light on this issue. Since differences between modalities are mainly present at early stages of perceptual processing, with representations retaining more of the properties and dimensions of the proximal stimuli (the sensory signals), more and stronger differences in the applicability of Gestalt principles between modalities suggest that their related mechanisms operate at early stages of perceptual processing. If, in contrast, a Gestalt principle operates at higher levels of perceptual processing, with representations in which the sensory aspects of the proximal stimuli are already filtered out in order to achieve more invariant representations of the perceived objects, no differences between modalities should be present. In this paper we will focus on the Gestalt principle of grouping by proximity, and its applicability in the haptic modality.

The haptic sense, in relation to the Gestalt theory and its grouping principles, is particularly interesting because of its active and multisensory nature. One of the major differences between visual and haptic perceptual processes is the position and number of the perceptual sensors. In the visual modality the sensors are in a more or less fixed relative position to each other. In contrast, if we explore an object by touch, we do not only use the sensors in our skin, but we need to move our hands and digits in order to obtain information related to the type of material we are dealing with or the shape characteristics of the object [7]. The knowledge about the position of our hands and digits (proprioception) has to be combined with the tactile input on the skin (touch) in order to, for example, determine the location of an object or its edges. This process takes time [8] and involves distinct areas in the brain [9]. Because of these obvious differences between vision and haptics, it remains an open question whether or not the Gestalt principle of grouping by proximity also applies in haptics. Depending on the stage of haptic processing, the Gestalt principle of proximity could operate at two distinct levels: proximity on the skin (somatotopic proximity; before integration of touch with proprioception) or proximity in external space (spatial proximity; after integration of touch with proprioception). In a recent study [10] we tried to unravel this issue by varying both somatotopic and spatial distance between target and distractor pairs in a haptic search task. The hypothesis was that if the Gestalt law of proximity would affect search by grouping of distractor pairs, we should obtain shorter search times for target pairs that were closer together. However, we did not find an effect for spatial proximity nor for somatotopic proximity. We proposed in the discussion of that paper that this was due to the nature of the task, in which target pairs were completely defined by spatial orientation such that no need arose for processing proximity information. Earlier studies indeed showed that participants could use proximity information in haptic tasks if explicitly asked by the experimenter how many groups of items are present in a display. Under such instructions both spatial [11] and temporal [12] proximity had an effect. Studies using explicit instructions to group tactile stimuli show that participants have the capacity to exploit haptic proximity in the first place; however, they do not directly speak to the issue of whether proximity is being used spontaneously in the context of haptic object recognition, like it does vision. Another potential limitation of these earlier studies was that haptic trials were used in combination with visual conditions within the same experiment, which in our view encourages heavier reliance on visual mediation (e.g. [13]). A danger of this procedure is that visual perception informs and drives grouping of haptic stimuli.

Our goal in the current study was to extend the investigation of the applicability of the Gestalt principle of proximity to the haptic modality to *spontaneous* grouping processes serving the detection of object contours. We felt that this promised more direct evidence along the lines of similarities between vision and haptics. To do so, we refrained from asking participants explicitly to group stimuli. Instead, we used a contour detection task, where participants simply had to indicate whether or not a contour was present in the display. We defined the contour by closer proximity and even distribution of its elements compared to the background elements that were randomly distributed and on average separated by a larger distance. It is known that Gestalt principles influence the detectability of a contour in visual displays. The Gestalt principle of proximity is the most straightforward: The closer the elements are together the easier it is to detect the contour (e.g. [14]). Because this is such a strong finding in the visual domain, we decided to use this type of task and translate it to the haptic domain. We varied the proximity of the contour and background elements, so that proximity was the defining characteristic of the target. Moreover, we decided that the contour is present in only 50% of the trials, so that an absent response in one of the trials would represent no grouping, while a present response would represent spontaneous grouping.

**Figure 1:**
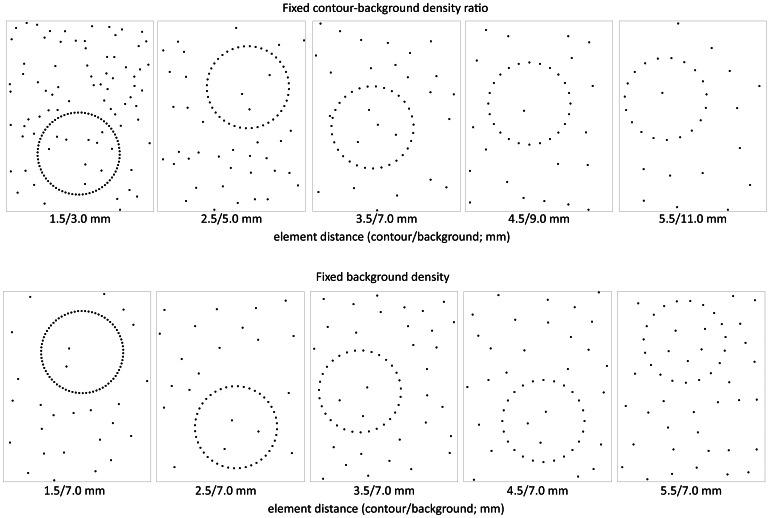
Cutouts and downscaled examples of the stimuli of all conditions. Contour present examples shown only. The top row represents examples of the fixed contour/background ratio condition and the bottom row examples of the fixed background condition.

The contour was defined by a closer proximity of elements as compared to the background and we varied the proximity of both the contour and the background elements (Figure 1). By doing so, our target is directly mapped to the output of the mechanoreceptors in the skin (especially type I mechanoreceptors: Meissner corpuscles -FAI- and Merkel disks -SAI-; [15]), hereby circumventing any visual mediation. We used two experimental conditions: We defined proximity in relative *and* in absolute measures compared to the background (distractors), by keeping either the ratio between contour and background constant, or by keeping the background constant. If contour proximity alone determines detection time and accuracy, we expect to find similar results independent of background density. However, if, in contrast, the determining factor would be the detectability of the contour in the background, the ratio between contour and background is more important, and we would expect different results for these two conditions. Thus, by using this approach we target the process of grouping by proximity at a tactile level.

A second and related question is whether the number of sensors used influences grouping. It is known from previous research that the input on the digits cannot always be processed in parallel [16], [17], [18], unless what is felt under the finger pads can be integrated, or grouped, into a whole. Thus, if the input of the background can be integrated across digits, we should find faster detection times and lower error rates when more digits are used. This should be the case not only because the number of sensors that can be used simultaneously when more digits are used, but also because the contour is more salient when the background (distractor) elements can be grouped together ([c.f. visual search [19], [20], [21]).

In sum, we investigate whether grouping by proximity influences the detectability of haptic contours. Our paradigm focuses on grouping by proximity based on responses of mechanoreceptors by avoiding explicit grouping instructions. Through our systematic variation of contour/background density ratios, we can assess the effects of proximity grouping independent of other potential detectability effects. Finally, we contrasted haptic exploration with single index digits and all digits of the dominant hand to determine a potential contribution of grouping of background elements to contour detection. We hypothesized that closer proximity of contour elements support grouping, resulting in larger proportions of correct detection and shorter exploration times, even if detectability effects were controlled for and independent of the number of digits used.

## Results

The Shapiro-Wilk test for normality of the data showed that for the majority of the conditions the data differed significantly from a normal distribution for both proportion correct (all 40 conditions p<.05) and for exploration times (19 out of 40 conditions p<.05). We therefore opted for nonparametric tests for the analyses.

The proportion correct trials, reported in contour absent trials, were very high: .99±.01 and .99±.01 for one finger and .99±.01 and .99±.01 for all fingers; for fixed background and fixed ratio respectively. Proportion correct for contour present trials were significantly lower than contour absent trials (.83±.03 and.91±.02 for one finger and .83±.03 and .88±.03 for all fingers; for fixed background and fixed ratio respectively; all Z>2.32, all p<.05 (related samples Wilcoxon signed rank test)). Errors in target present conditions consisted mainly of misses (99.93% of the total number of errors), hardly any false alarms were registered (0.07%; the contour was not touched yet, but where the participant indicated that one was present). As one would expect based on serial search models (e.g. Overvliet et al., 2007b), in both conditions exploration times were significantly lower in the contour present trials (10.99±1.40 s and 11.28±1.01 s for one finger and 10.19±2.36 s and 9.47±1.61 s for all fingers; fixed background and fixed contour respectively) as compared to contour absent trials (23.95±3.95 s and 24.09±2.20 s for one finger and 19.63±3.46 s and 18.60±2.62 s for all fingers; fixed background and fixed contour respectively; all Z>2.80, all p<.01 (related samples Wilcoxon Signed Rank Test). Because our hypothesis that closer proximity of contour elements supports grouping, resulting in larger proportions of correct detection and shorter exploration times, is only formulated for contour present trials, we will omit contour absent trials from the remainder of the analysis.

**Figure 2:**
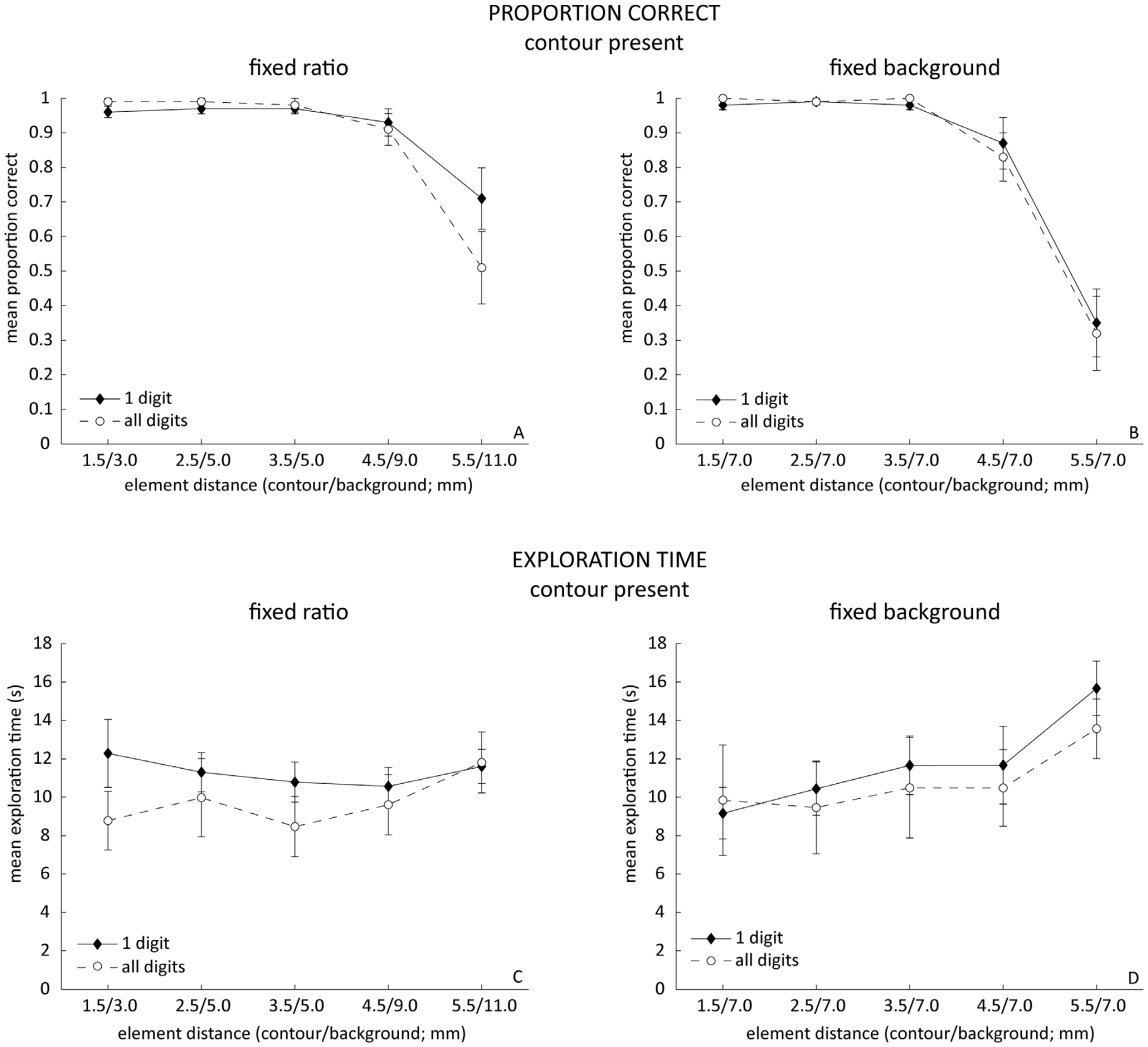
Results of the experiment. (A–B): Mean proportion correct of trials for fixed contour/background ratio and fixed background conditions for all densities. (C–D): Mean exploration time for correct trials only, for fixed contour/background ratio and fixed background conditions for all densities. Error bars represent the standard error of the mean over participants.

The proportion correct trials and exploration times for all conditions are shown in Figure 2. We first tested which data points for proportion correct were significantly different from 1, to avoid testing ceiling effects. In the fixed ratio condition 1.5 mm/3.0 mm for one finger and 5.5 mm/11.0 mm for both finger conditions and in the fixed background condition 4.5 mm/7.0 mm for all fingers and 5.5 mm/11.0 mm for both finger conditions were significantly different from 1 (all p<.05; one sample Wilcoxon Signed Rank Test). We therefore decided to focus our further analysis for proportion correct to the 4.5 mm and 5.5 mm contours. A Generalized Linear Model with predictors contour density (4.5 or 5.5 mm), contour-background ratio condition (fixed ratio or fixed background), and number of digits (one or all) showed main effects for contour density (Wald χ^2^_df = 1_ = 590.05, p<.0001) and for contour-background ratio (Wald χ^2^_df = 1_ = 9.53, p<.01), and a marginally significant interaction between these two factors (Wald χ^2^_df = 1_ = 3.36, p = .067). This shows that 5.5 mm contours more difficult to detect compared to 4.5 mm contours. Although this is the case in both fixed ratio and fixed background conditions, the latter one resulted in lower percentages correct as compared to the fixed ratio condition.

Another Generalized Linear Model on the exploration times of the correct trials, with predictors contour density (1.5, 2.5, 3.5, 4.5, 5.5 mm), contour-background condition (fixed ratio, fixed background) and number of digits used (one, all) did not reveal any significant effect.

The experimenter monitored the movements of the participants and observed that all participants were scanning the display in a systematic fashion by zigzagging diagonally, horizontally or vertically. As soon as they hit the contour, the participants always checked whether it was the contour by tracing it with their index finger, independent of finger condition (one vs. all). The average exploration times as reported above are thus rather conservative, but contour tracing was observed as just a quick check and should by far not be the main portion of the exploration times as reported. This is also confirmed by the finding that contour absent trials are roughly twice as long as compared to contour present trials, which is expected based on haptic serial search literature (e.g. [16]).

## Discussion

In this study we investigated the applicability of the Gestalt principle of grouping by proximity in the haptic modality. To this end we used a haptic contour detection task, in which contour elements differed from background elements in terms of their closer, spatial proximity. As predicted, closer proximity of contour elements generally lead to higher detection rates and shorter exploration times. Grouping by proximity effects were equally robust, for varying contour background density ratios as well as across conditions in which we kept this ratio constant. At the same time, the main effect was robust independent of the number of digits used. We argue that this is evidence for spontaneous haptic grouping by proximity in a study design that controls for explicit grouping instructions and discriminability effects.

The strongest support for this claim comes from our finding that proximity effects were present in the fixed background condition *as well as* in the fixed ratio condition. Note that when target dots were separated by 5.5 mm contour detection dropped significantly in all conditions suggesting that grouping was very weak or non- existent for such larger distances. We argue that this points to a type of threshold for this type of haptic grouping phenomenon. In other words, if certain conditions are met and grouping occurs, a salient contour pops out of the background, and will almost always be detected. Assuming that a critical threshold for grouping may be around 5 mm is actually quite plausible, because at that distance one cannot perceive more than two dots simultaneously with the sensitive part of one’s finger pad. Feeling a minimum of three continuous dots simultaneously on the finger pad may be critical in order to group them and establish a difference with respect to the background dots. Another possibility is that when distances between dots are larger than about 4 to 5 mm, the type II mechanoreceptors start responding in an organized fashion [15], their signals might interfere with the output of the type I receptors in SI and therefore introducing more errors in contour detection. Given our very limited knowledge about interactions between different receptor types in tactile perception this interpretation remains speculative.

The finding that the use of all digits did not lead to differences in exploration time compared to the single-digit condition may seem surprising. Arguably, the area that can be explored simultaneously with multiple fingers is larger, which should increase chances of hitting contour elements within the larger display. Moreover, in everyday life we typically benefit from bimanual and multi-finger exploration (cf. [22]). However, previous studies by the first author showed that input from different fingers is processed in a serial fashion [23] unless it is integrated into one object or surface. Such serial processing may even lead to slowing down of exploration speed [17].

Previous studies already pointed towards grouping by proximity in haptic tasks. Chang, Nesbitt and Wilkins [11] asked participants to explicitly state how many groups of items they (haptically) perceived. They intermingled haptic and visual exploration trials such that a general strategy could have been adopted, instead of a modality specific strategy, which may have involved visual mediation in the haptic trials. We do not completely rule out the possibility of visual mediation in our current experimental design, but we reason that the influence should be less prominent when only haptic conditions are measured. We do by no means want to imply that visual mediation does not play a role in haptic processing; evidence from our own studies [24] in fact supports the classic view [13]. In an earlier study [10], when instructional effects were controlled for and the tasks were exclusively haptic, we did find grouping by similarity, but not by proximity, possibly because the target was defined by similarity and not by proximity cues. We claim that the current study provides evidence for the *spontaneous* operation of the Gestalt principle of grouping by proximity in haptic processing. The evidence from the present study, notably the limits for grouping we identified, suggests that Gestalt processes are not limited to early stages of *visual* processing. We argue that the proximity principle also operates at early stages of *haptic* processing. The claim that Gestalt processes also operate at later stages in both modalities when more general representations have been formed remains a valid option to be investigated in future studies.

## Methods

### Ethics Statement

The study was conducted in line with the ethical principles regarding research with human participants as specified in The Code of Ethics of the World Medical Association (Declaration of Helsinki). The study was approved by the Medical Ethical Committee of the University Hospital Gasthuisberg (Leuven), and the participants gave written informed consent before starting the experiment.

### Participants

Ten participants took part in the study (mean age 23.1±4.36, 9 right handed, 8 female). We measured both tactile sensitivity (mean score 4.6±.52; maximum score is 5) and moving and static 2-point discrimination (mean score 2.7±.48 mm and 2.8±.63 mm respectively) by using the Touch-Test® Sensory Evaluators and the Touch-Test® Two-Point Discriminator (North Coast Medical, Inc., USA). None of the participants had a score below “normal” as indicated by the Touch-Test® manufacturer.

### Stimuli

We constructed the stimuli using ZY-TEX2® Swell Paper (Zychem Ltd., Cheshire, England) by using the ZY®Fuse Heater. The stimulus area was 19 by 19 cm. We defined the contour as a regular distribution of dots on a circle with a diameter of 4 cm. The contour could be placed anywhere on the stimulus area as long as it had a minimal distance to the border of 1 cm of the stimulus area. After placing the contour, the background was filled with random dots. For each experimental condition there were 20 unique stimuli: 10 with a contour, and 10 without a contour (just background). These were individually generated by using the GERT (Grouping Elements Rendering Toolbox) as developed by Demeyer and Machilsen [25]. The diameter of the dots was 0.7 mm and they protruded about 1 mm from the surface of the swell paper. Target absent trials were randomly placed background dots not containing a contour. Note that circular contours also have good continuation, but because this good continuation is only an added value after the differences in proximity between the dots on the contour and the dots in the background have been detected, proximity is the primary grouping cue here. Moreover, in the visual literature nowadays, the term “good continuation” is preserved for conditions where proximity and density are controlled or the elements themselves are oriented (e.g., line segments or Gabor patches).

We defined the contour densities in accordance with the spatial resolving capacities of the mechanoreceptors in the skin of the finger pad as measured by Philips, Johansson and Johnson [15]. They found that when the distance between 2 dots in the stimulus was below 1.5 mm none of the mechanoreceptors had a clear response pattern, while above 1.5 mm type I mechanoreceptors could spatially resolve them with peak responses at 2.5 mm for SAI and 3.5 mm for FAI. Type II mechanoreceptors start responding in patterns after 3–4 mm spatial distance between dots. However, they do not have a peak response at any spatial distance as measured by Philips et al. Another factor that is important for our stimulus construction is the size of the receptive fields of the different mechanoreceptors: While the type II mechanoreceptors have quite large receptive fields with fuzzy boundaries (59–101 mm^2^ for SAII and FAII respectively), type I mechanoreceptors have small receptive fields with sharp boundaries (11.0–12.6 mm^2^ for SAI and FAI receptors respectively) [26]. We therefore decided to use several dot spacings for the contour that cover all these characteristics, which thus include all the distances of the contours. Because velocity of scanning does not influence spatial resolving capacities of the mechanoreceptors [15], participants were allowed to explore the displays in their own preferred speed.

We manipulated two variables: the ratio between contour and background (fixed ratio vs. fixed background), and the number of digits that the participants were allowed to use to explore the displays (one vs. all), which resulted in four experimental blocks. In two of the blocks of trials the ratio between the contour and background was constant (always 2∶1), in the other blocks the background density was always the same (average separation of 7.0 mm between the dots), while the contour density varied (see Figure 1 for examples of the stimuli). In 50% of the trials there was no contour present. This resulted in a total of 4 blocks of 100 trials for each participant (20 trials for each condition as shown in Figure 1). We counterbalanced the order in which the blocks of trials were tested based on the number of fingers that were used and randomized the order of the two different contour/background conditions. Moreover, within each block all trials were randomized.

### Procedure

The four blocks of trials were run in four separate sessions that lasted about one hour each. In half of the sessions the participants were allowed to use just one digit (the index finger of their dominant hand) and in the other half they were free to use all digits of their dominant hand or even their full hand to explore the stimuli (we observed that participants generally used 3 to 5 fingers). For one of each two sessions we kept the contour – background ratio constant, while for the other of each two sessions we kept the background constant, while the contour varied in density. The order of the use of digits was counterbalanced over the participants, within these two sets of conditions the contour-background ratio was also counterbalanced. At the beginning of the first sessions the experimenter measured participants’ tactile acuity (as described in the Participants section) and asked them to read and sign the informed consent form. The experimenter then seated the participants in the experimental set-up and blindfolded them. In each trial the experimenter put the index finger of the dominant hand of the participant in the centre of the stimulus. When the starting signal sounded, participants started exploring the display and as soon as they found a contour they pressed a pedal with their left foot, when they thought there was no contour present in the display, they had to press a pedal with their right foot. The participants were told that the contour that they had to search for had always the same diameter of 4 cm, and that it would be present in 50% of the trials. No feedback was given during the course of the experiment. Each session started with 10 practice trials, in which feedback was given.
